# 

**Published:** 1861

**Authors:** 


New Series, Vol. 4, Nos. 10 & 11. Whole Series, Vol. 18, Nos. 10 & 11.

### THE CHICAGO

### MEDICAL JOURNAL.

DANIEL BRAINARD, M. D.,
J. ADAMS ALLEN, M. D.,
Editors and Proprietors.

OCTOBER & NOVEMBER, 1861.

3

CHICAGO:
WM. PIGOTT, BOOK AND JOB PRINTER, 130 CLARK STREET.
1861.

